# m^6^A RNA methylation counteracts dark-induced leaf senescence in Arabidopsis

**DOI:** 10.1093/plphys/kiad660

**Published:** 2023-12-12

**Authors:** Arsheed H Sheikh, Naheed Tabassum, Anamika Rawat, Marilia Almeida Trapp, Kashif Nawaz, Heribert Hirt

**Affiliations:** Center for Desert Agriculture, Division of Biological and Environmental Sciences and Engineering, King Abdullah University of Science and Technology, Thuwal 23955-6900, Saudi Arabia; Center for Desert Agriculture, Division of Biological and Environmental Sciences and Engineering, King Abdullah University of Science and Technology, Thuwal 23955-6900, Saudi Arabia; Center for Desert Agriculture, Division of Biological and Environmental Sciences and Engineering, King Abdullah University of Science and Technology, Thuwal 23955-6900, Saudi Arabia; Center for Desert Agriculture, Division of Biological and Environmental Sciences and Engineering, King Abdullah University of Science and Technology, Thuwal 23955-6900, Saudi Arabia; Center for Desert Agriculture, Division of Biological and Environmental Sciences and Engineering, King Abdullah University of Science and Technology, Thuwal 23955-6900, Saudi Arabia; Center for Desert Agriculture, Division of Biological and Environmental Sciences and Engineering, King Abdullah University of Science and Technology, Thuwal 23955-6900, Saudi Arabia

## Abstract

Senescence is an important physiological process which directly affects many agronomic traits in plants. Senescence induces chlorophyll degradation, phytohormone changes, cellular structure damage, and altered gene regulation. Although these physiological outputs are well defined, the molecular mechanisms employed are not known. Using dark-induced leaf senescence (DILS) as the experimental system, we investigated the role of N^6^-methyladenosine (m^6^A) mRNA methylation during senescence in Arabidopsis (*Arabidopsis thaliana*). Plants compromised in m^6^A machinery components like METHYLTRANSFERASE A (*mta* mutant) and VIRILIZER1 (*vir-1* mutant) showed an enhanced DILS phenotype. This was accompanied by compromised chloroplast and photosynthesis performance in *mta* as well as accumulation of senescence-promoting camalexin and phytohormone jasmonic acid after dark treatment. m^6^A levels increased during DILS and destabilized senescence-related transcripts thereby preventing premature aging. Due to inefficient decay, senescence-related transcripts like *ORESARA1* (*ORE1*), *SENESCENCE-ASSOCIATED GENE 21* (*SAG21*), *NAC-like*, *activated by AP3/PI* (*NAP*), and *NONYELLOWING 1* (*NYE1*) over-accumulated in *mta* thereby causing accelerated senescence during DILS. Overall, our data propose that m^6^A modification is involved in regulating the biological response to senescence in plants, providing targets for engineering stress tolerance of crops.

## Teaser

RNA m^6^A modification prevents premature aging in plants by destabilizing senescence-related transcripts.

## Introduction

Senescence in plants is a highly coordinated process which is triggered in response to both internal and external environmental cues. The primary internal trigger for plant senescence is age dependent whereby a highly synchronized senescence response is induced during later phases of plant development. On the other hand, harsh environmental stresses can also trigger premature senescence in affected plant tissues (Guo and Gan 2005). One of the key external triggers of senescence is exposure to prolonged darkness, a process termed dark-induced senescence (DIS) (Liebsch and Keech 2016). In addition to DIS, prolonged darkness can cause other developmental defects of skotomorphogenic structures such as, apical hook, elongated hypocotyls, and shortened roots in plants (Deepika et al. 2020). Given the importance of leaves in photosynthesis, light deprivation or darkness can show remarkable senescence phenotypes in leaves, a process termed dark-induced leaf senescence (DILS). It is important to understand premature senescence as it has a detrimental effect on the normal life span of plants thereby reducing the biomass of plants. This also makes it of high economic relevance as DIS can strongly influence post-harvest shelf-life and yield in agriculturally relevant crop plants (Sade et al. 2018).

The major physiological and genetic changes caused by DILS are altered hormonal dynamics, gene regulation, chloroplast integrity, and chlorophyll degradation (Sobieszczuk-Nowicka et al. 2018). DILS is a catabolic process where cellular organelles and biomolecules are degraded. The most striking feature of DILS is the yellowing caused by the breakdown of chlorophyll (Hörtensteiner 2006). This is achieved through the regulation of both biosynthesis and/or degradation of chlorophyll. For example, Chl-b is converted to Chl-a before it is channeled to the degradation pathway. Chl-b degradation is initiated by key reductase enzymes likes NONYELLOW COLORING 1 (NYC1) and NYC1-like (NOL) (Sato et al. 2009) while Chl-a catabolism is initiated by NONYELLOWINGs/STAY-GREENs (NYEs/SGRs) enzymes (Sakuraba et al. 2015). At the same time senescence causes downregulation of photosynthetic genes like *RIBULOSE BISPHOSPHATE CARBOXYLASE SMALL CHAIN* (*RBCS*) *and CHLOROPHYLL A/B BINDING PROTEIN1* (*CAB1*) (Park et al. 1998).

The catabolism of chlorophyll is accompanied by the structural degradation of chloroplasts. In later phases of senescence perturbed stacking of thylakoids is observed while the number and size of plastoglobules (thylakoid-associated lipid droplets) increases (Tamary et al. 2019). Also, the repression of the master regulators of chloroplast maintenance *GOLDEN-LIKE* (*GLK1* and *GLK2*) is observed (Waters et al. 2009). The combined effect of chloroplast and chlorophyll degradation leads to reduced photosynthetic efficiency and enhanced ion leakage of the plant tissues (Wojciechowska et al. 2018).

Plant hormones play a central role in senescence. Phytohormones like abscisic acid (ABA), ethylene, jasmonic acid (JA), and salicylic acid (SA) promote senescence, while cytokinins and auxin inhibit this process (Woo et al. 2019). For example, JASMONATE-ZIM-DOMAIN PROTEIN 7 (JAZ7), a JA repressor protein, is a negative regulator of DILS (Yu et al. 2016). Dark stress induces ABA accumulation through the induction of ABA biosynthetic genes which consequently promote DILS by chlorophyll degradation (Mao et al. 2017).

Transcriptional changes are one of the major regulators of both age-dependent and DIS. For example, transcriptome experiments carried out in Arabidopsis, rice (*Oryza sativa*), barley (*Hordeum vulgare*), and desiccation tolerant *Haberlea* have revealed that up to 30% of the genes show expression changes during age-dependent senescence and DILS (Woo et al. 2016; Durgud et al. 2018; Sobieszczuk-Nowicka et al. 2018; Gad et al. 2021). The hallmark of the senescence program is the induction of expression of thousands of *SENESCENCE-ASSOCIATED GENEs* (*SAG*s). Transcription factor-mediated regulation of *SAG* expression has emerged as a critical regulatory mechanism in the leaf senescence process. Noticeably, TF families like NACs and WRKYs are among the main TFs regulating DILS. One of the positive regulators of DILS is the master NAC TF called *ORESARA1* (*ORE1/ANAC092*) leading to induction of many core *SAG*s (Woo et al. 2019). Also, the *SAG*s involved in chlorophyll catabolism like *STAY-GREEN 1* (*SGR1*) and *NONYELLOW COLORING 1* (*NYC1*) are regulated by ORE1 and phytochrome-interacting factors (PIFs) (Song et al. 2014). Interestingly, ORE1 antagonizes the transcriptional activity of key chloroplast development and activity maintainer protein GLK1/GLK2 (Golden 2-like Transcription factor 1/2) (Waters et al. 2009). TFs of the WRKY family are involved in senescence and include WRKY6, WRKY22, WRKY53, WRKY54, WRKY70, and WRKY75 (Guo et al. 2021).

The regulation of gene expression can be achieved at epigenetic and epi-transcriptomic levels. Among the many cellular mechanisms that regulate mRNA fate, m^6^A has emerged as a major regulator of mRNA processing, localization, stability, and translatability (Arribas-Hernández and Brodersen 2020). N^6^-methyladenosine (m^6^A) is the most prevalent internal covalent mRNA modification in eukaryotic transcriptomes. In Arabidopsis, the m^6^A writer complex consists of METHYLTRANSFERASE A (MTA), METHYLTRANSFERASE B (MTB), and FKBP INTERACTING PROTEIN 37 (FIP37), which all have highly conserved mammalian putative orthologs (Methyltransferase Like 3 *(*METTL3), Methyltransferase Like 14 (METTL14), and Wilm’s tumor 1 (WTAP) associated protein, respectively) (Reichel et al. 2019). In addition, two important proteins VIRILIZER (VIR) and HAKAI were found to be part of this complex where the downregulation of these proteins led to reduced relative m^6^A levels (Růžička et al. 2017). m^6^A is a dynamic process and the m^6^A mark can be removed by erasers like Alpha-ketoglutarate-dependent dioxygenase (AlkB) and AlkB-homology (AlkBH) family proteins. m^6^A-decorated sites are directly recognized and bound by reader proteins that contain methyl-binding aromatic pockets (YTH domain) named as EVOLUTIONARILY CONSERVED C-TERMINAL REGION (ECTs) in Arabidopsis (Reichel et al. 2019). One of the key functions of m^6^A in plants is the regulation of mRNA stability being either a stabilizing or a destabilizing mark in different physiological conditions (Shen et al. 2016; Duan et al. 2017; Anderson et al. 2018). However, m^6^A is generally found as a destabilizing mark in animal systems by facilitating RNA decay usually via reader proteins (Lee et al. 2020).

The regulation of gene expression during stress-induced senescence is also achieved by various mechanisms like epigenetic changes by H3K27me3 demethylation, miRNAs, and posttranslational mechanisms like ubiquitination (Guo et al. 2021). However, we lack an understanding of gene expression regulation during DILS by posttranscriptional changes like m^6^A. Here, we report that m^6^A is enriched in plants upon dark stress and global m^6^A levels increase during DILS. Consequently, the Arabidopsis m^6^A mutant in METHYLTRANSFERASE A (*mta)* shows a pronounced DILS phenotype when compared to wild-type plants. The senescence-related transcripts accumulate in *mta.* m^6^A decreases the stability of senescence-related transcripts thereby countering dark stress-induced senescence. Overall, our data propose m^6^A modification to be implicated in regulating the biological response of plants to early senescence.

## Results

### *mta* mutant exhibits accelerated senescence phenotype

**Figure 1.**
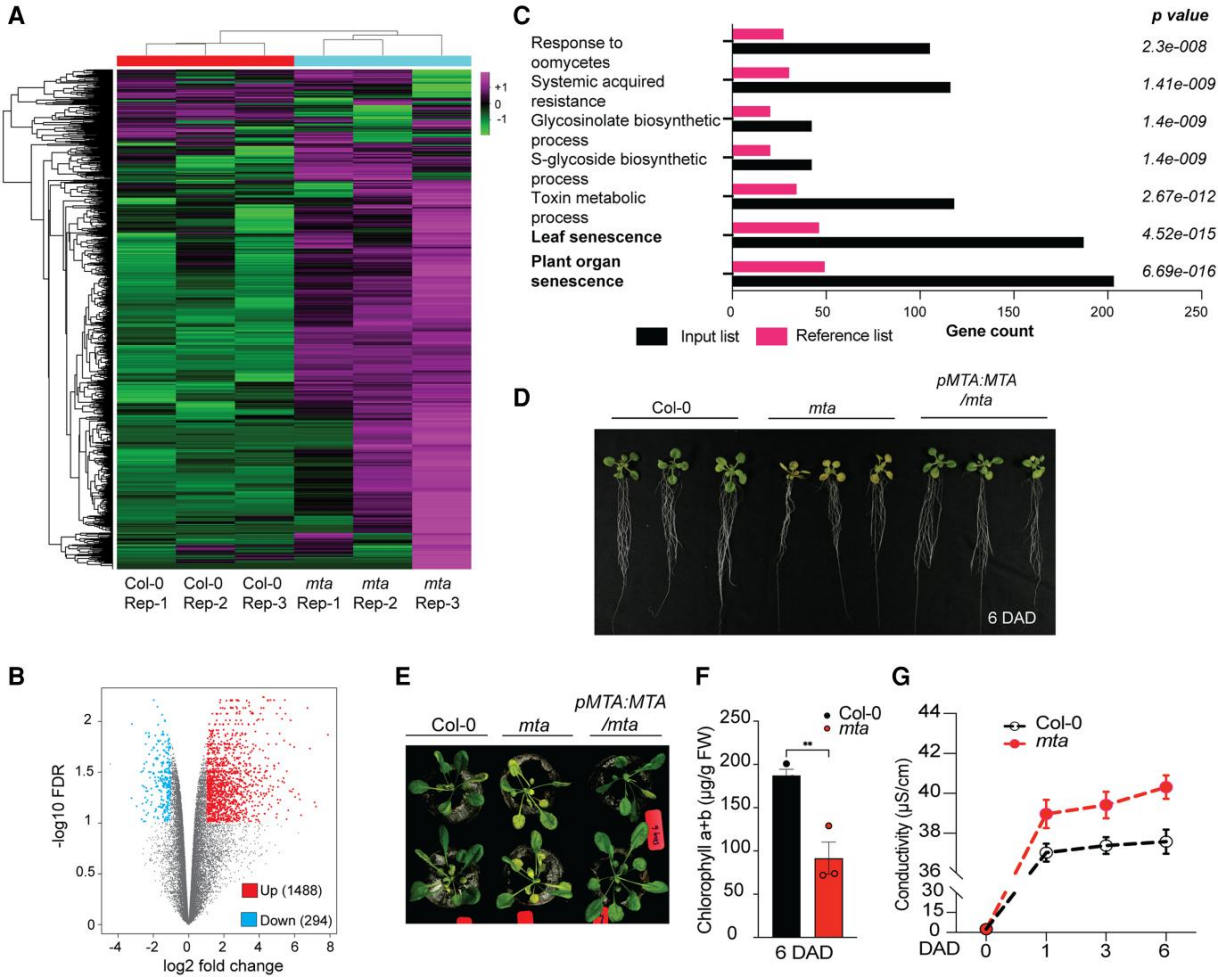
Accelerated senescence phenotype of mta mutant upon DILS. **A)** Heatmap showing significant (2.0 FC, FDR *P*_adj_ < 0.01) DEGs across the three biological replicates in Col-0 and *mta*. The colors represent as the *z*-score transformed values of DEGs across all samples. **B)** Volcano plot showing the number of upregulated and downregulated genes in *mta* as compared to Col-0. **C)** GO analysis showing significant enrichment of biological pathways in *mta* mutant. **D)** Comparison of leaf senescence between 2-wk-old seedlings of Col-0, *mta*, and complementation line *pMTA::MTA/mta* plants at 6 days after dark (DAD). **E)** The DILS phenotype comparison at 6 DAD between adult plants of Col-0, *mta*, and *pMTA::MTA/mta* plants grown in jiffy pots for 4 wk. **F)** Comparison of the total chlorophyll content (chl a + b) of 2-wk-old leaves between Col-0 and *mta* plants at 6 DAD. Values are presented as the mean ± SEM (*n* = 3) (two-tailed paired Student’s *t*-test, **P ≤ 0.01). **G)** Ion leakage represented as conductivity from leaf discs of Col-0 and *mta* recorded at 1, 3, and 6 d upon dark treatment. Values are presented as the mean ± SEM.

To understand the role of m^6^A in gene regulation, we performed a transcriptomic analysis in the mutant of the main m^6^A writer MTA. We used the well-defined m^6^A deficient mutant of Arabidopsis *mta ABI3::MTA* (onwards called *mta*) in which MTA is driven by ABI3 promoter which enables its expression only during germination to allow growth of the otherwise embryo lethal null mutant (Bodi et al. 2012). Comparison of RNA-seq of 4-wk-old Col-0 and *mta* plants showed dynamic gene expression profiles (Fig. 1A). We observed that 1,488 genes were upregulated while 294 were downregulated in *mta* as compared to Col-0 (Fig. 1B). Gene ontology (GO) analysis revealed a significant enrichment of transcripts related to leaf senescence (Fig. 1C). Consistently, *mta* mutant shows an early senescence phenotype (Supplementary Fig. S1A). To understand the underlying molecular details, we exploited the DILS which is a well-established proxy to study senescence (Hao et al. 2022). We observed that *mta* seedlings shows enhanced sensitivity to DILS when compared to Col-0 with an increased yellowing of leaves during dark treatment (Fig. 1D). The *mta* phenotype was restored to WT in *pMTA::MTA-YFP* where *mta* is complemented with the genomic MTA (AT4G10760) locus (Fig. 1D). The enhanced DILS phenotype of *mta* was maintained from seedling to adult developmental stages (Fig. 1E). Also, the chlorophyll content of *mta* was significantly lower than that of wild-type plants during DILS, while no significant changes were observed under control conditions (Fig. 1F; Supplementary Fig. S1B). Consistently, the *mta* also displayed a higher ion leakage than Col-0 plants (Fig. 1G).

### *mta* mutant plants have enhanced levels of DILS-related transcripts

**Figure 2.**
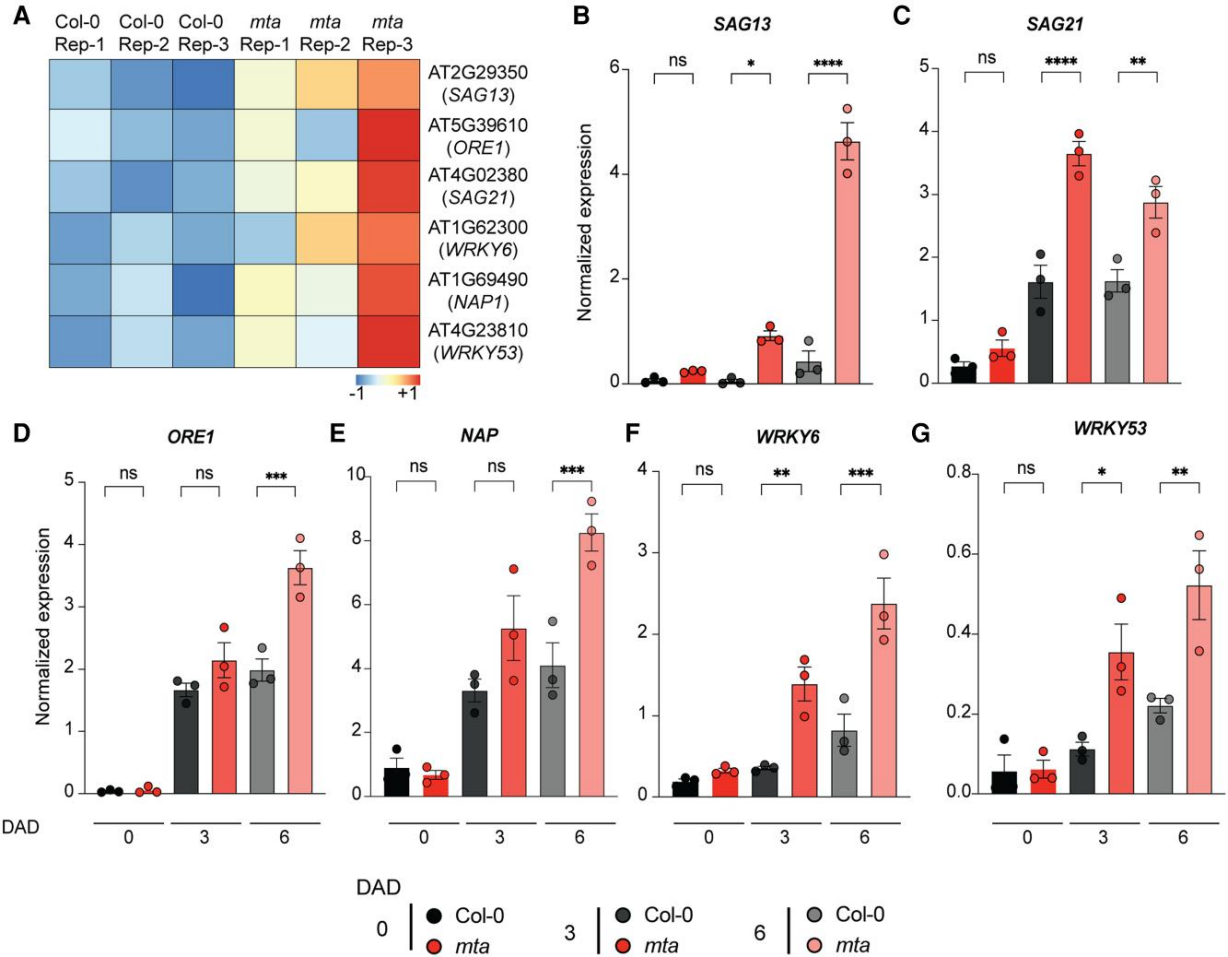
*mta* mutant plants have elevated transcript levels of senescence responsive genes. **A)** Heatmap showing significant (2.0 FC, FDR *P*_adj_ < 0.01) DEGs across the three biological replicates in Col-0 and *mta*. The colors are representing the *z*-score transformed values of DEGs across all samples. **B and C)** Expression levels of key senescence marker genes *SAG13***(B)**, *SAG21***(C)** in Col-0 and *mta* seedlings upon dark treatment for 3 and 6 d. **D and E)** Expression levels of NAC TF master regulators of senescence *ORE1***(D)**, *NAP***(E)** in Col-0 and *mta* seedlings upon dark treatment for 3 and 6 d. **F and G)** Expression levels of WRKY TF regulators of senescence *WRKY6***(F)**, *WRKY53***(G)** in Col-0 and *mta* seedlings upon dark treatment for 3 and 6 d. All the results shown are normalized to *UBQ* expression as an internal control. Values are presented as the mean ± SEM (*n* = 3). *P < 0.05, **P < 0.01, ***P < 0.001, ****P < 0.0001, multiple one-way ANOVA with Sidak’s test. ns, not significant; DAD, days after dark.

As we observed a significant enrichment of senescence process in GO analysis, we investigated the expression of key senescence-related genes as shown in the heat map (Fig. 2A). We next tested the expression of these genes in the DILS setup using seedlings dark treated for 3 or 6 d. We observed significantly higher levels of *SAG13* and *SAG21* at 3 and 6 d after dark in *mta* mutant as compared to Col-0 (Fig. 2, B and C). We observed a similar expression pattern of other senescence-related gene *SAG12* (Supplementary Fig. S1C). Next, we sought to investigate the expression of SAG master regulators like NAC and WRKY TFs. We observed a higher expression of *ORE1*, *NAP*, *WRKY6*, and *WRKY53* in *mta* after 3 and 6 DAD (days after dark) (Fig. 2, D to G). As senescence induces ROS production, we measured the expression of two known ROS-regulated genes *OXIDATIVE SIGNAL INDUCIBLE1* (*OXI1*) and *Thioredoxin h5* (*Trx-h5*) (Beaugelin et al. 2019). We observed massive transcript levels of *OXI1* in *mta* at 3 DAD while *Trx-h5* was higher in *mta* at 6 DAD (Supplementary Fig. S1, D and E). In addition, we used another mutant of the m^6^A writer machinery *vir-1* to study the DILS phenotype. Dot blot analysis showed that *vir-1* has lower global m^6^A levels than Col-0 (Supplementary Fig. S1F). Consistently, we observed an enhanced DILS phenotype in *vir-1* mutant (Supplementary Fig. S1G) and enhanced levels of *SAG21*, *SAG113*, *WRKY6*, and *WRKY53* transcripts (Supplementary Fig. S1, H to K). This shows the DILS phenotype is regulated by the m^6^A machinery.

### Chloroplast activity is compromised in *mta* mutant during DILS

**Figure 3.**
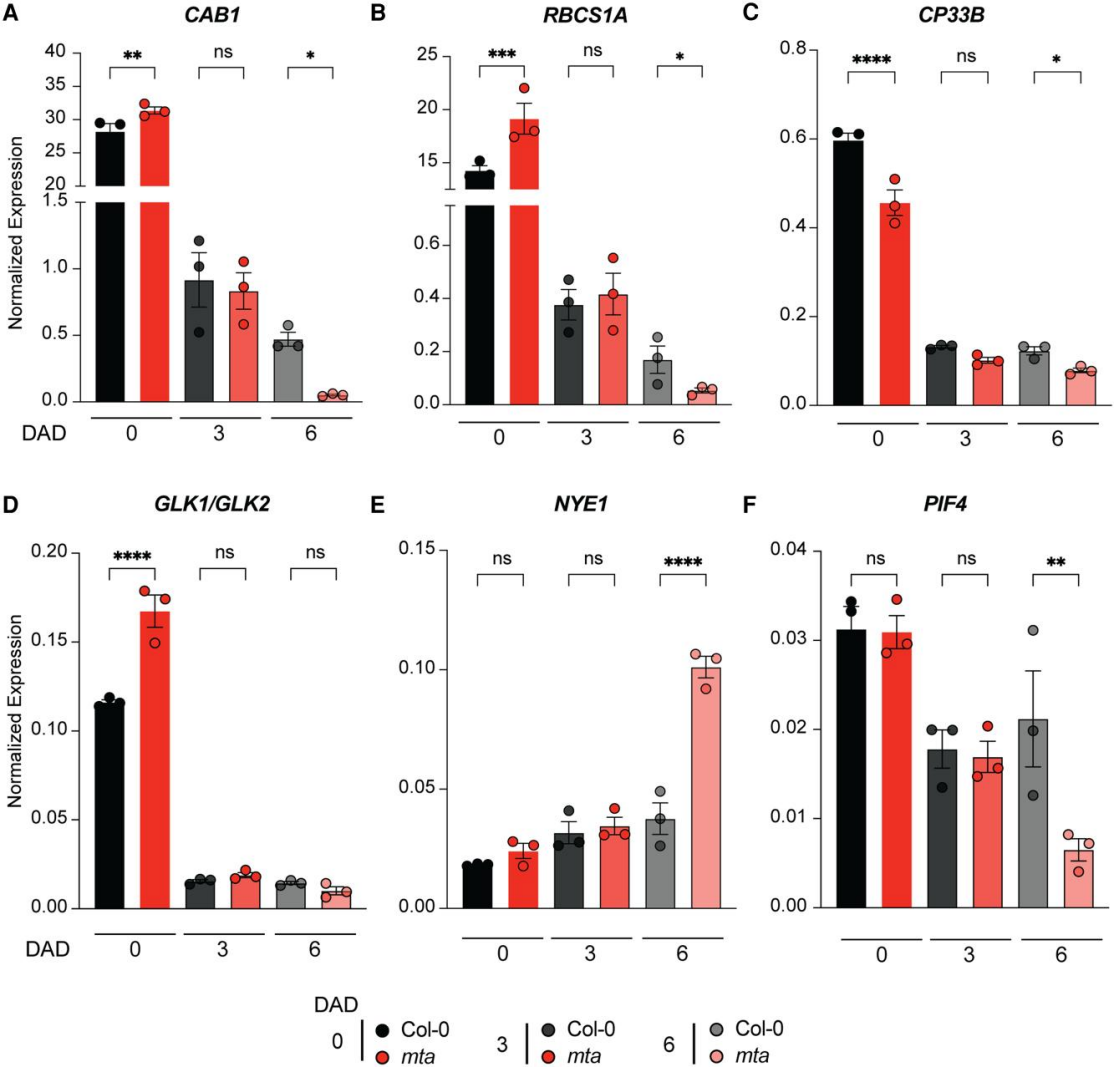
*mta* mutant plants exhibit compromised chloroplast activity during DILS. **A–C)**, Expression levels of key photosynthetic genes *CAB1***(A)**, *RBCS1A***(B)**, and *CP33B***(C)** in Col-0 and *mta* seedlings upon dark treatment for 3 and 6 d. **D)** Expression levels of crucial chloroplast development and activity maintaining marker gene *GLK1/2* in Col-0 and *mta* seedlings upon dark treatment for 3 and 6 d. **E)** Expression levels of chlorophyll degradation and catabolism regulator *NYE1* in Col-0 and *mta* seedlings upon dark treatment for 3 and 6 d. **F)** Expression levels of phytochrome gene regulating senescence *PIF4* in Col-0 and *mta* seedlings upon dark treatment for 3 and 6 d. All the results shown were normalized to *UBQ* expression as an internal control. Values are presented as the mean ± SEM (*n* = 3). *P < 0.05, **P < 0.01, ***P < 0.001, ****P < 0.0001, multiple one-way ANOVA with Sidak’s test. ns, not significant; DAD, days after dark.

During senescence, the expression of chloroplast function and photosynthesis maintenance marker genes is massively regulated. We investigated the expression of two key photosynthetic genes, CHLOROPHYLL A/B-BINDING PROTEIN 1 (*CAB1*) and RIBULOSE BISPHOSPHATE CARBOXYLASE SMALL CHAIN 1A (*RBCS1A*) and a stroma localized PSBA RNA-binding protein *CHLOROPLAST RIBONUCLEOPROTEIN 33B (CP33B)* during dark stress. We observed that the transcript levels of these genes were significantly downregulated in *mta* than in Col-0 at 6 d of dark stress while subtle changes were observed at 3 DAD (Fig. 3, A to C). Similarly, the transcript levels of *GLK1/GLK2* (Golden 2-like Transcription factor 1/2), the crucial chloroplast development and activity maintaining marker genes dropped faster in dark-stressed *mta* as compared to wild-type plants given its expression was higher in *mta* at control conditions (Fig. 3D). On the other hand, the levels of chlorophyll degradation regulator *NYE1* (Nonyellowing 1, also called *SGR1*) which promotes chlorophyll catabolism was higher in *mta* than Col-0 at day 6 DAD (Fig. 3E). PIFs are known to regulate the expression of these genes during DILS (Song et al. 2014). Interestingly we observed lower levels of *PIF4* in *mta* than Col-0 at 6 DAD (Fig. 3F).

### m^6^A deficiency confers accelerated chloroplast and photosystem damage during dark stress

**Figure 4.**
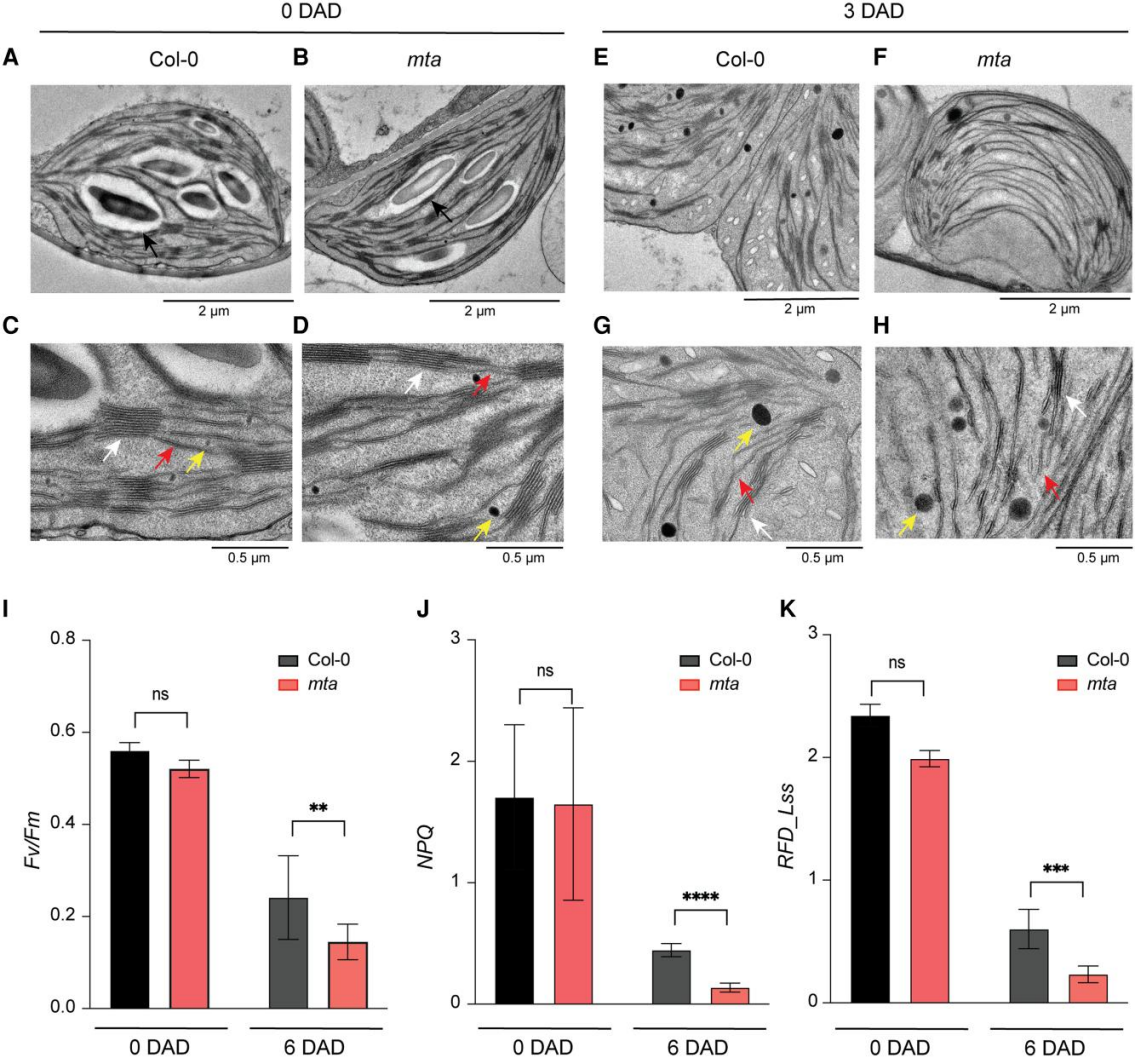
Accelerated chloroplast and photosystem damage in *mta* mutant plants during DILS. **A and B)** TEM images of chloroplasts in Col-0 (**A**) and *mta***(B)** seedlings under normal conditions (0 DAD). Chloroplasts with lenticular shape and big starch grains are shown by black arrows. (Scale bar = 2 *μ*m.) **C and D)** TEM imaging of chloroplasts from Col-0 and *mta* showing typical internal chloroplast structures at 0 DAD. White arrows show intact thylakoid systems stacked as grana. Red arrows show grana connected by stroma lamellae and yellow arrow shows tiny plastoglobules. (Scale bar = 0.5 *μ*m.) **E and F)** TEM imaging of chloroplast ultrastructure in Col-0 **(E)** and *mta***(F)** after 3 d of dark treatment (3 DAD). The altered chloroplast structures by dark treatment are shown with *mta* having an almost spherical chloroplast and no starch grains. (Scale bar = 2 *μ*m.) **G and H)** TEM imaging of chloroplasts from Col-0 and *mta* at 3 DAD. White arrows show dismantled thylakoid membranes with almost no intact grana. Red arrows show disintegrated stroma lamellae and yellow arrow shows big plastoglobules. (Scale bar = 0.5 *μ*m.) **I–K)***F*_v_/*F*_m_ ratios showing the maximum quantum efficiency of PSII **(I)**, non-photochemical quenching (NPQ) **(J)**, and relative fluorescence decline (RFD_Lss) **(K)** in Col-0 and *mta* plants before (0 DAD) and after (6 DAD) treatment. Values were counted by using Photon System Instruments, PSI system. Data presented as mean ± SEM, *P < 0.05, **P < 0.01, ***P < 0.001, ****P < 0.0001, multiple one-way ANOVA with Sidak’s test. ns, not significant; DAD, days after dark.

Leaf yellowing is the most striking DILS phenotype caused by damaged chloroplasts harboring the photosynthetic machinery of the plant cell. To test whether accelerated DILS in *mta* was accompanied by the disintegration of chloroplast structures, we performed a cytological analysis of chloroplasts using TEM imaging. In control conditions, Col-0 and *mta* chloroplasts showed typical structures with visible outer and inner membranes and intact thylakoid systems (Fig. 4, A to D). However, *mta* chloroplasts changed from lenticular to spherical shapes and showed enhanced swirling of thylakoids as compared to Col-0 after 3 d of dark treatment (Fig. 4, E and F). The disintegration of the chloroplasts was visibly more obvious in dark stressed *mta* (Supplementary Fig. S2A). While Col-0 still had loosely stacked grana, *mta* plants had dismantled thylakoids with almost no intact grana (Fig. 4, G and H). We also observed bigger plastoglobules in *mta* than Col-0 after DILS (Fig. 4, G and H, Supplementary Fig. S2B).

To study the effects of m^6^A on the photosynthesis machinery during dark stress, we performed high throughput phenotyping of Col-0 and *mta* plants using PlantScreen System (Photon System Instruments, PSI). After confirming the DILS phenotype in a PSI compatible tray system (Supplementary Fig. S2C), we recorded the various photosynthetic parameters in 2-wk-old plants before and after 6 d of constitutive dark stress. During DILS, the maximum quantum efficiency of PSII photochemistry (*F*_v_/*F*_m_), indicating the plant photosynthetic efficiency, had significantly decreased in *mta* plants as compared to Col-0 (Fig. 4I). Nonphotochemical quenching (NPQ), indicating the heat loss from PSII, was also significantly lower in *mta* plants compared to Col-0 (Fig. 4J). Consistently, the other vital parameters like relative fluorescence decline (RFD) and max quantum yield (QYmax) were also lower in *mta* than Col-0 plants (Fig. 4K, Supplementary Fig. S2D).

### *mta* exhibits a dynamic hormone profile during DILS

**Figure 5.**
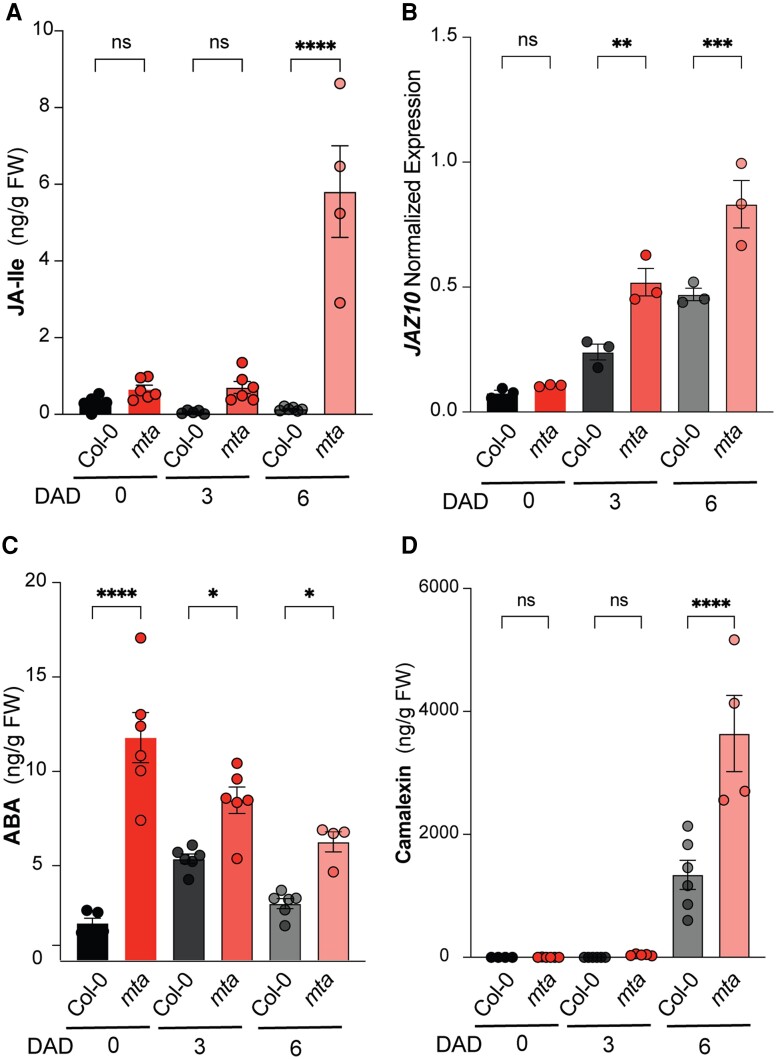
*mta* mutant plants exhibit altered JA and ABA hormone profiles during DILS. **A)** Quantification of JA intermediate (JA-Ile) levels (ng/g fresh weight) in Col-0 and *mta* seedlings upon dark treatment for 3 and 6 d. Data is presented as mean ± SEM. ****P < 0.0001, multiple one-way ANOVA with Sidak’s test. ns, not significant; JA-Ile, jasmonate-isoleucine. **B)** Expression levels of JA-mediated senescence regulator gene *JAZ10* in Col-0 and *mta* seedlings dark treated for 3 and 6 d. Data is presented as mean ± SEM (*n* = 3). **P < 0.01, ***P < 0.001, multiple one-way ANOVA with Sidak’s test. ns, not significant. **C and D)** Quantification of ABA and Camalexin levels in Col-0 and *mta* seedlings dark treated for 3 and 6 d. Data is presented as mean ± SEM. *P < 0.05, ****P < 0.0001, multiple one-way ANOVA with Sidak’s test. DAD, days after dark.

As changes in the plant physiology are governed by key hormones during DILS (Guo and Gan 2005), we determined levels of some of the key senescence-related phytohormones. We observed increased JA-Ile and JA levels in *mta* plants at 3 DAD, which further accumulated at 6 d of darkness (Fig. 5A, Supplementary Fig. S3A). These changes in JA levels were accompanied by an increase in transcript levels of *JAZ10,* a marker of DILS (Fig. 5B). Interestingly, increased ABA levels were already observed in *mta* in control conditions (Fig. 5C). Although ABA levels dropped after dark stress in *mta* plants, they were still higher than in Col-0 after dark treatment (Fig. 5C). Since ethylene is a prominent senescence promoting hormone, we also tested and observed higher levels of the ethylene signaling marker gene *ETHYLENE RESPONSE 2* (*ETR2*) in *mta* than Col-0 during DILS (Supplementary Fig. S3B). Since phytoalexins are known to be involved in premature leaf senescence (Pegadaraju et al. 2005), we also determined camalexin levels. After 6 d of dark, we also observed significantly higher camalexin levels in *mta* than in Col-0 (Fig. 5D), further establishing a role of m^6^A in DILS.

### m^6^A levels and the m^6^A machinery is dynamically regulated during dark stress

**Figure 6.**
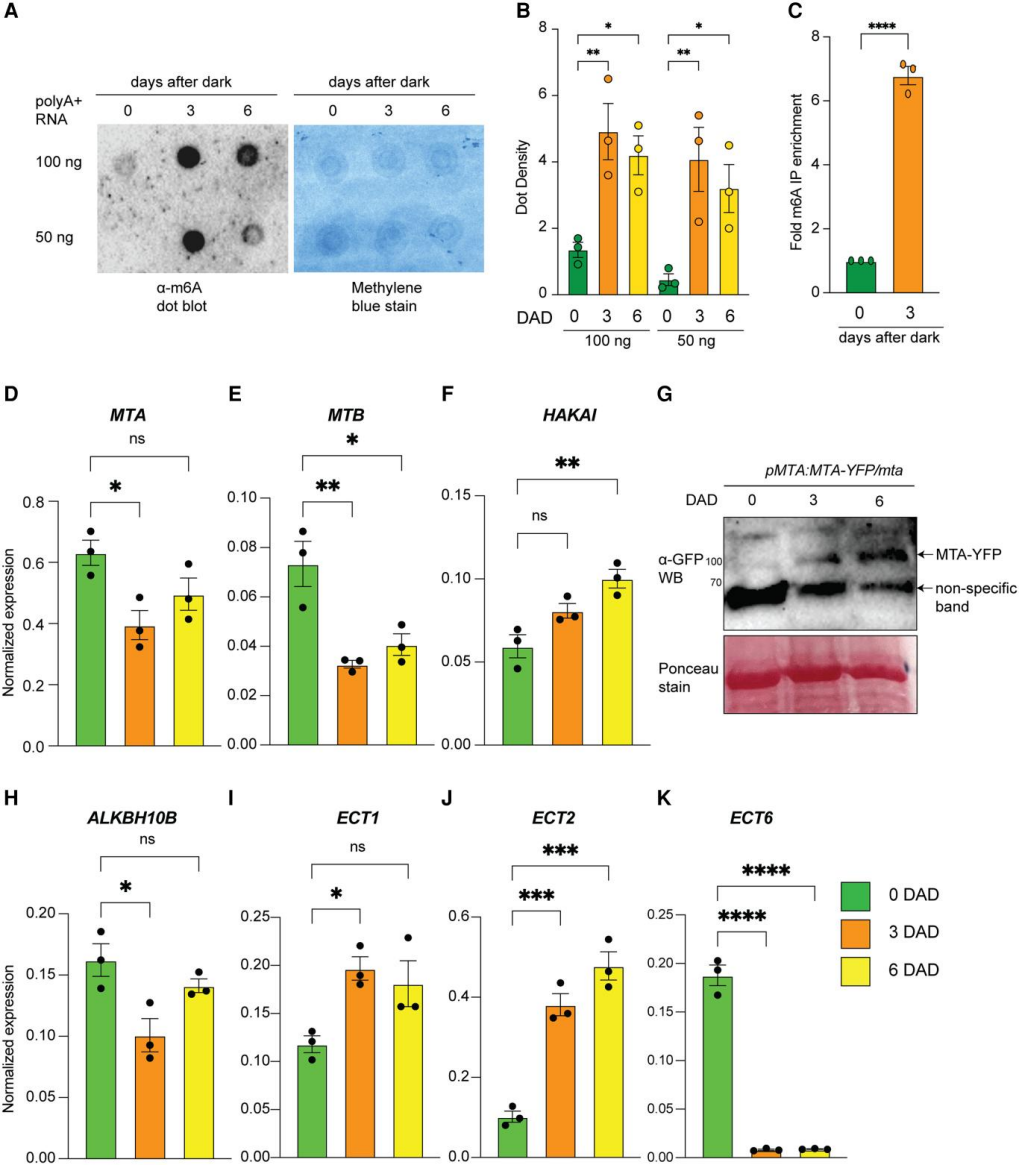
Dynamic regulation of the m^6^A machinery during DILS. **A)** Dot blot assay showing the levels of m^6^A in poly(A^+^) mRNA isolated from Col-0 seedlings at 3 and 6 DAD treatment. Methylene blue staining represents the loading control. **B)** Quantification of m^6^A levels by dot density. 3 *µ*L dots of 100 and 50 ng poly(A^+^) from the same replicate are blotted onto the nylon membrane. *n* = 3 biological replicates. Data presented as mean ± SEM, *P < 0.05, **P < 0.01, multiple one-way ANOVA with Sidak’s test. **C)** Fold enrichment of m^6^A immunoprecipitation relative to control (0 DAD) performed from poly(A^+^) mRNA isolated from 75 *μ*g of total RNA. Data presented as mean ± SEM, ****P < 0.0001, Student’s two-tailed *t*-test. **D to F)** Expression levels of core Arabidopsis m^6^A writer genes *MTA***(D)**, *MTB* (**E**), and *HAKAI***(F)** in Col-0 seedlings upon dark treatment for 3 and 6 d. Data presented as mean ± SEM. **G)** Western blot showing the protein levels of MTA in *pMTA:MTA-YFP/mta* complementation plant line at 0, 3, and 6 DAD. Ponceau stain shows the loading control. **H)** Expression levels of one of the main Arabidopsis m^6^A eraser genes *ALKBH10B* in Col-0 seedlings at 0, 3, and 6 DAD. **I to K)** Expression levels of some of the key Arabidopsis m^6^A reader genes *ECT1***(I)**, *ECT2***(J)**, and *ECT6***(K)** in Col-0 seedlings at 0, 3, and 6 DAD. All the results RT-qPCR results shown were normalized to *UBQ* expression as an internal control. Values are presented as the mean ± SEM (*n* = 3). *P < 0.05, **P < 0.01, ***P < 0.001, ****P < 0.0001, multiple one-way ANOVA with Sidak’s test. ns, not significant; DAD, days after dark.

As we observed an accelerated senescence phenotype in *mta* and *vir-1* mutants, we were interested to examine the changes in m^6^A levels during DILS. We observed an increase in the global levels of m^6^A after 3 and 6 d of darkness (Fig. 6, A and B). By performing immunoprecipitation from equal amounts of poly(A^+^)-enriched mRNA with m^6^A antibody, we observed an approximately 7-fold increase in the m^6^A-IP of RNA in dark-treated samples compared to control seedlings (Fig. 6C). We next investigated the expression of several components of the m^6^A machinery. We observed a decrease in transcript levels of *MTA* and *MTB,* two main m^6^A writer complex components (Fig. 6, D and E), but increased transcript levels of *HAKAI,* and subtle but insignificant changes in *FIP37* and *VIR1* levels were observed (Fig. 6F, Supplementary Fig. S4, A and B). However, the plants showed increased MTA protein levels after 3 and 6 d of dark stress (Fig. 6G). Decreased transcript levels for the eraser *ALKBH10B* were seen (Fig. 6H), but a stark upregulation of the m^6^A readers *ECT1* and *ECT2* at 3 and 6 DAD in wild-type plants (Fig. 6, I and J). In contrast, transcript levels of the m^6^A readers *ECT4*, *ECT6*, and *ECT8* strongly decreased (Fig. 6K, Supplementary Fig. S4, C and D), whereas those of *CLEAVAGE AND POLYADENYLATION SPECIFICITY FACTOR 30* (*CPSF30*) remained unaffected during DILS (Supplementary Fig. S4E). As the expression of the m^6^A reader proteins was strongly affected during dark, we examined the DILS phenotype in *ect2ect4* mutant plants. We observed an enhanced senescence in *ect2ect4* mutant, which was however less severe than for the *mta* mutant (Supplementary Fig. S4F). The results suggest that ECTs also play a role in the m^6^A response to dark stress.

### m^6^A machinery counteracts DILS by facilitating the decay of senescence-related transcripts

**Figure 7.**
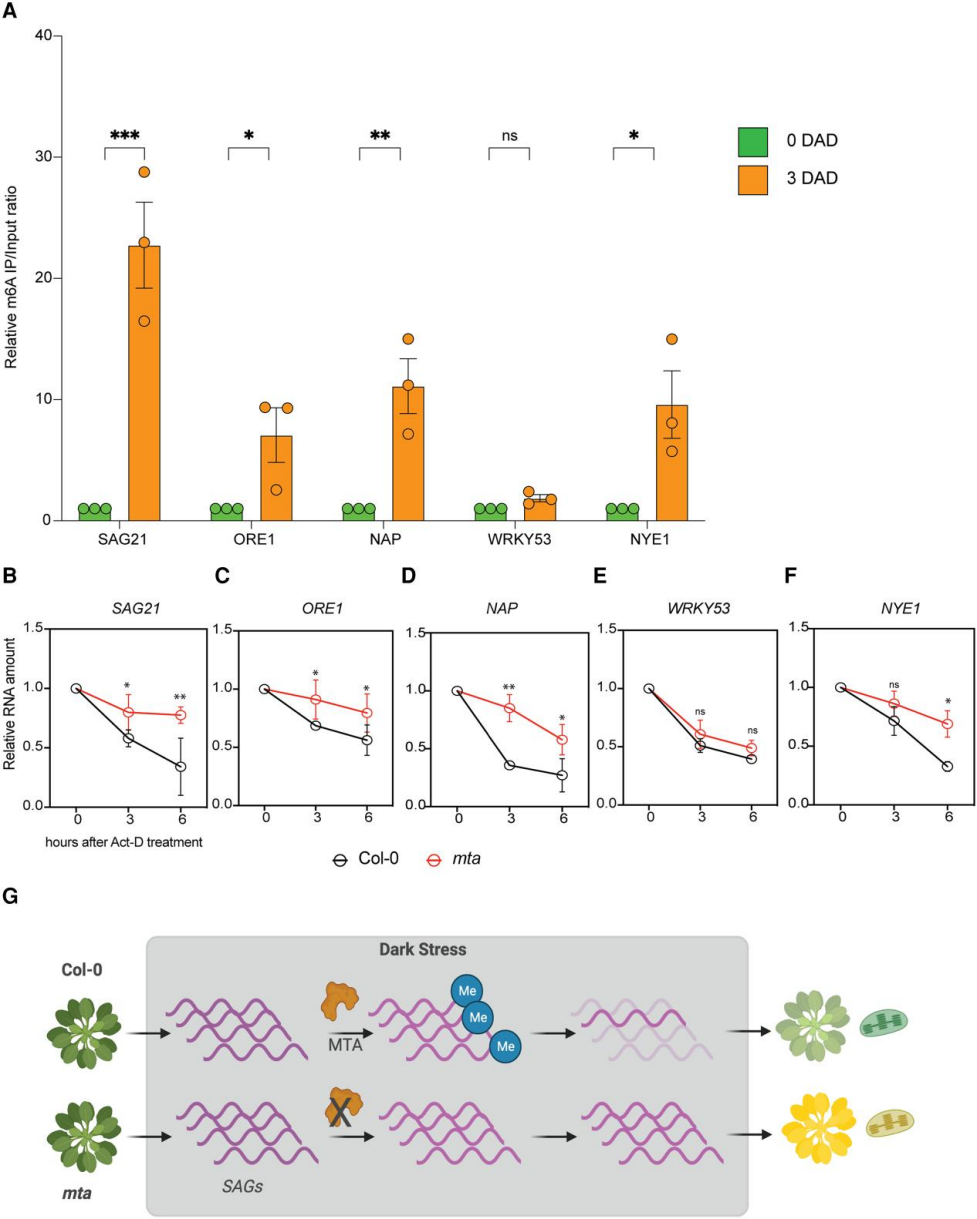
m^6^A regulates the abundance of senescence-related transcripts. **A)** m^6^A MeRIP of some of the key differentially regulated transcripts during DILS. Data are represented as relative IP/input ratio where IP/input at 3 DAD is normalized to IP/input at 0 DAD in Col-0 seedlings. Values are presented as the mean ± SEM (*n* = 3). *P < 0.05, **P < 0.01, ***P < 0.001, multiple one-way ANOVA with Sidak’s test. ns, not significant, DAD, days after dark. **B–F)** Actinomycin D RNA decay assay of 2-wk-old Col-0 and *mta* seedlings kept in dark for 3 d and then treated with a cocktail of 30 *μ*g mL^−1^ of Actinomycin D and 10 *μ*g mL^−1^ cordycepin and kept in constant light. Samples were harvested at 0 (immediately after Act-D treatment), 3 and 6 h in light. Relative transcript levels of *SAG21***(B)**, *ORE1***(C)**, *NAP***(D)**, *WRKY53***(E)**, and *NYE1***(F)** were quantified using RT-qPCR. Data are represented as relative RNA amount where RNA levels at 0 time point is considered 100%. Values are presented as the mean ± SD (*n* = 3). *P < 0.05, **P < 0.01, Paired student *t*-test. ns, not significant; DAD, days after dark. **G)** Model where DILS triggered senescence-related transcript abundance is regulated by the m^6^A machinery by facilitating their efficient decay. Compared to Col-0, in *mta* mutant plants, SAGs over accumulate leading to enhanced DILS.

As we observed increased m^6^A levels and *ECT2* transcript levels during DILS in wild-type plants (Figs. 1A and 6G), we reasoned that m^6^A might prevent DILS by facilitating the decay of *SAG*s. To this end, we performed mRNA immunoprecipitation with m^6^A antibody (m6A Me-RIP) and examined the m^6^A enrichment of several senescence-related transcripts. We observed that transcripts of some of the key senesce markers like *SAG21* were highly enriched for m^6^A at 3 d after dark treatment (Fig. 7A). Also, transcripts of the NAC TF master regulators *ORE1* and *NAP* showed significant m^6^A enrichment, while no m^6^A enrichment was observed for the WRKY TF *WRKY53* (Fig. 7A). We also observed m^6^A enrichment in transcripts of the chloroplast targeted senescence marker *NYE1* (Fig. 7A).

Next, we sought to investigate the effect of m^6^A on these transcripts. Given the effect of m^6^A on RNA stability in eukaryotes (Lee et al. 2020), we hypothesized that m^6^A might contribute to transcript level changes via alteration of RNA decay. To this end, we performed mRNA decay experiments in dark stressed plants which were subsequently treated with Actinomycin-D for transcription inhibition and kept in constitutive light to reverse the DIS response. We observed an accelerated decrease of *SAG21* and *ORE1* transcripts in Col-0 compared to *mta* plants (Fig. 7, B and C). A similar behavior of accelerated decrease was observed in *NAP* and *NYE1* transcripts in Col-0 compared to *mta* plants (Fig. 7, D and F) while no significant changes were observed in decay rates of *WRKY53* (Fig. 7E).

Taken together these results decipher a critical role of the m^6^A machinery in counteracting DIS by partly regulating their stability (Fig. 7G).

## Discussion

m^6^A regulates critical physiological processes including development and stress responses in plants (Reichel et al. 2019; Arribas-Hernández and Brodersen 2020). Dark stress induces senescence in plants which is detrimental for total biomass and yield of plants. However, the molecular mechanisms to adapt or resist to stress-induced senescence are not known. Here we provide insights into the role of m^6^A in the premature senescence response of plants. We show that m^6^A levels increase during dark treatment and that *mta* mutant plants are hyper-sensitive to DILS. In wild-type plants, m^6^A effectively destabilizes senescence-related transcripts during DILS onset while *mta* mutant plants over-accumulate these transcripts leading to an enhanced DILS phenotype. These data suggest a critical involvement of m^6^A in the modulation of RNA levels during dark stress (Fig. 7E) and are in agreement with the study where METTL3 (human homolog of MTA) is indispensable for preventing human mesenchymal stem cells from accelerated senescence (Wu et al. 2020). However, reduced m^6^A modification levels were observed in senescing hMSCs (Wu et al. 2020) which were accompanied by the downregulation of *METTL3*, similar to our observation for *MTA* expression. On the other hand, we observed increased MTA protein levels upon dark stress, which could explain the higher m^6^A levels in Arabidopsis during dark treatment. Also, m^6^A led to stabilization of *MINICHROMOSOME INSTABILITY 12* (*MIS12*) mRNA which prevents hMSCs from accelerated senescence, while we observed a contrasting mechanism where m^6^A destabilizes senescence enhancing mRNAs (like *SAG*s) culminating in a similar physiological outcome as in hMSCs. The direct role of m^6^A machinery in regulating crop development like fruit ripening process has been discussed recently (Zhou et al. 2022). For example, tomato (*Solanum lycopersicum*) m^6^A demethylase SlALKBH2 mediates the removal of m^6^A on key ripening-promoting DNA demethylase *DEMETER-LIKE PROTEIN 2* (*SlDML2)* transcripts. This increases the transcript stability thereby facilitating fruit ripening (Zhou et al. 2019). In contrast, the strawberry (*Fragaria virginiana*) m^6^A methyltransferase FvMTA-mediated incorporation of m^6^A on key ABA genes which either increases their stability or translation, thereby facilitating fruit ripening (Zhou et al. 2021). As fruit ripening is a close-knit process with senescence, it will be interesting to study the role of m^6^A on general or stress-induced senescence in crops plants in the future. In addition to directly regulating the stability of senescence-related genes, lack of m^6^A can also disturb the pri-miRNA processing and lead to a reduction in global miRNA levels (Bhat et al. 2020). These changes in miRNA levels can then regulate the accumulation of senescence-related transcripts like *ORE1* which is a known target of miRNA164 (Li et al. 2013).

In addition to stability, m^6^A is also involved in regulating many other mRNA processes like localization, splicing, and translation, so it is possible that alternative regulatory mechanisms might be employed to prevent DILS. We also observed an intermediate DILS phenotype in mutant plants of the m^6^A readers ECT2 ECT4, suggesting involvement of m^6^A readers in regulating the dark stress response. Similar to ECT2 in Arabidopsis, the Drosophila (*Drosophila melanogaster*) homolog *YTH domain containing 1* (*Ythdc1*) showed increased expression in brain upon heat shock (Perlegos et al. 2022). YTHDC family members like YTHDC1, 2, and 3 are well known to regulate RNA stability via various mechanisms (Morris et al. 2021). We observed that m^6^A can regulate the levels of both nuclear, cytosolic as well as chloroplast localized transcripts. Interestingly, up to 40% of m^6^A-modified transcripts are associated with chloroplasts (Luo et al. 2014). Out of the many m^6^A decorated senescence-related mRNAs (Fig. 7), *SAG21* was already reported as an m^6^A target by the METTL6 homolog FIONA1 (FIO1) in plants (Sun et al. 2022). Interestingly, *fio1* mutant plants display an enhanced age-related senescence phenotype, suggesting there might exist a coordinated response of multiple m^6^A methyltransferases in senescence regulation.

The changes in transcript levels observed for several senescence-related genes in *mta* plants could be an indirect effect of m^6^A deficiency. For example, the master regulator TF ORE1 influences the expression of a number of senescence-related genes (Liebsch and Keech 2016). In this regard, the m^6^A-mediated regulation of *ORE1* could also indirectly affect the expression of *SAG*s like *SAG12*, *SAG13*, and *SEN4*. Similarly, the differential accumulation of hormone levels observed in *mta* plants could also play an important role in coordinating the senescence process. The two prominent GO categories in *mta* plants, namely response to oomycete and glucosinolate biosynthesis (Fig. 1C), could be a reflection of higher JA and camalexin levels. In addition to promote senescence, it will be interesting to test *mta* mutant for bacterial and fungal immunity response as these genetic networks may be connected (Woo et al. 2019; Zhang et al. 2020). Also, the higher levels of ABA in *mta* plants under control conditions could provide a trigger for accelerated senescence during DILS as ABA levels are known to regulate leaf senescence (Liebsch and Keech 2016; Sakuraba et al. 2020). However, the reduced levels of ABA observed after DILS in our study could be a result of feedback repression of *NAP* expression on ABA biosynthesis as shown in rice (Liang et al. 2014).

On the other hand, JA levels slightly increased at day 3 of dark treatment, while its levels massively accumulated in *mta* after prolonged darkness of 6 d (Fig. 5). As MeJA is known to regulate the expression of photosynthesis genes like *CAB* and *RBCS*, the rapid increase in JA levels at day 6 could explain their downregulation in *mta* at 6 DAD and not at 3 DAD (Fig. 3, A and B; Qi et al. 2015).

The changes in chloroplast cytology like damaged thylakoid structure and enlarged plastoglobules observed in Arabidopsis (Fig. 4) were similar to the effects observed in barley under prolonged dark treatment (Sobieszczuk-Nowicka et al. 2018). Also, active modifications of DNA and RNA were observed during DILS in barley suggesting the role of epigenetic and epi-transcriptomic regulation during DILS (Sobieszczuk-Nowicka et al. 2018; Rudy et al. 2022). It will be interesting to investigate how the m^6^A machinery regulates senescence-related responses in crops. Overall, engineering of the m^6^A machinery of crops might provide a way to minimize crop yield loss to early senescence.

## Conclusions

Our work unravels the role of m^6^A in DILS response. We suggest that the accumulation of senescence-related transcripts during DILS may require exquisite and dynamic posttranscriptional control. We provide evidence that m^6^A modification may be one mechanism that plants use to exert that control. m^6^A deposition thus modulates dark-induced stress response pathways by fine-tuning RNA decay of some selected transcripts. We showed that m^6^A modification of these key master transcripts could directly or indirectly regulate the overall senescence process. Our study expands the understanding of the cellular machineries which counteract early senescence and could provide a unique target for crop engineering to combat crop loss.

## Materials and methods

### Plant material and DILS treatment

Arabidopsis (*Arabidopsis thaliana*) ecotype Col-0 and *mta ABI3:MTA* (*mta*) were used in this study. *mta* mutant was generated as described earlier (Bodi et al. 2012). MTA complementation line was generated by Agrobacterium (*Agrobacterium tumefaciens*) mediated transformation of *mta* plants by MTA genomic locus cloned into pGWB440 vector.

### RNA sequencing

RNA sequencing of 4-wk-old adult Col-0 and *mta* plants was performed with three biological replicates. RNA from mature leaves was extracted using Nucleospin RNA plant kit (Macherey-Nagel) following the manufacturer’s recommendations. The quality and quantity of the RNA was assessed using Nanodrop-6000 spectrophotometer, 2100-Bioanalyzer (RNA integrity number greater than 8.0), and QubitTM 2.0 Fluorometer with the RNA BR assay kit (Invitrogen). By using Illumina TruSeq standard mRNA Library Preparation protocol, RNA-seq was performed as per manufacturer’s instructions for 50 base pair paired-end sequencing. Pooled libraries were sequenced using Illumina HiSeq 4,000 platform. After trimming and read alignment, DESeq2 was run with read counts to identify DEGs between the genotypes with FDR ≤ 0.01 (Love et al. 2014). Functional enrichment of DEGs was carried out with AgriGO using default settings (Tian et al. 2017).

### Dark-induced senescence assay

For DILS assays, seeds were surface sterilized and stratified at 4 °C for 3 d. Seedlings were then grown for 2 wk in large square Petri dishes containing 0.5× Murashige Skoog Basal Salts (Sigma #M6899), 0.5% (w/v) agar type E (Sigma #A4675), 0.05% (w/v) MES pH 5.7 (Sigma #M8250), at 16 h light/8 h dark, average lighting of 120 *μ*mol m^−2^ s^−1^, 22 °C day/20 °C night, 55% humidity. Plates were covered with aluminium foil to create complete darkness for 3 or 6 d. For DILS in pots, 4-wk-old adult plants grown in jiffy pots were covered with a lid and completely wrapped in aluminium foil to create constitutive darkness.

### Measurement of chlorophyll content

Chlorophyll content was determined as previously described (Lichtenthaler 1987). Briefly, the fresh leaves were weighed and ground in liquid nitrogen. Eighty percent (v/v) acetone (0.1 mL mg^−1^ leaf tissue) was added, and the samples were incubated at room temperature for 30 min with shaking. The samples were centrifuged, and the chlorophyll concentration of supernatant was calculated using a spectrophotometer (Tecan). The content was determined spectrophotometrically using the formula Chl (a + b) = 5.24*A*_664.2_ + 22.24*A*_648.6_ in μg mL^−1^, *A* is absorption at the indicated wavelength.

### Ion leakage assay

Leaf discs were incubated in 2.5 mM MES buffer containing 0.05% (v/v) Tween-20. The samples were incubated at 30 °C in dark for 6 d and readings were taken at 0, 1, 3, and 6 DAD using a Conductivity meter (Seven Excellence, Mettler Toledo). The data is represented as µS cm^−1^ of leaf disc.

### Total RNA and mRNA extraction from seedlings

Total RNA was extracted from the Arabidopsis seedlings grown on ½ MS with the Nucleospin RNA plant kit (Macherey-Nagel#740949) following the manufacturer’s recommendations. The quality and quantity of the RNA were assessed using Nanodrop-6000 spectrophotometer, 2100-Bioanalyzer (RNA integrity number greater than 8.0). poly(A^+^) + mRNA was isolated from 100 *µ*g of total RNA using Oligo-dT dynabeads from mRNA isolation kit (Thermo#61006).

### Dot blot assay

To determine relative abundance of m^6^A using membrane antibody-based detection, 100 and 50 ng of poly(A^+^) selected mRNA (described above) was spotted on SensiBlot plus Nylon membrane (Fermentas #M1002) and air dried for 5 min. The membrane was UV crosslinked using Stratalinker and blocked with PBST-5% (w/v) milk for 3 h. Membrane was incubated overnight with m^6^A antibody (abcam#15320, 1:2500), washed and subsequently incubated with goat antirabbit-HRP (Promega, 1:10,000). The membrane was developed using Immobilon Femto Western HRP substrate (Thermo #34094). The intensity of the dot blots was measured by using Image J software.

### m^6^A immunoprecipitation (MeRIP)

mRNA (1 *µ*g) was fragmented to 200 to 300 nucleotides by addition of 50 mM MgCl_2_ and incubated at 85 °C for 4 min. MeRIP was performed as previously described with several modifications (Wilson et al. 2020). Briefly, 25 *µ*L of protein G magnetic beads (Thermo Fisher Scientific) were washed twice with IP buffer (10 mM Tris-HCl pH 7.5, 150 mM NaCl, 0.1% (v/v) NP-40 in nuclease-free H_2_O) and then resuspended in 500 *µ*L of IP buffer. One microliter of anti-m^6^A antibody (New England Biolabs) was added at 4 °C for 4 h with constant shaking. The antibody–bead mixture was washed twice with IP buffer and resuspended in 300 *µ*L of the IP reaction mixture containing 1 *µ*g of fragmented mRNA, and 3 *µ*L of RNasin Plus RNase Inhibitor (Promega), and incubated overnight at 4 °C. After incubation the low/high-salt-washing method was applied: briefly the RNA reaction mixture was washed twice in 1,000 *µ*L of IP buffer, once in 1,000 *µ*L of low-salt IP buffer (50 mM NaCl, 10 mM Tris-HCl, pH 7.5, 0.1% (v/v) NP-40 in nuclease-free H_2_O), once in 1,000 *µ*L of high-salt IP buffer (500 mM NaCl, 10 mM Tris-HCl, pH 7.5, 0.1% (v/v) NP-40 in nuclease-free H_2_O) and once in 1,000 *µ*L of IP buffer for 2 min each at 4 °C. After washing, the m^6^A-enriched RNA was eluted from the beads in 200 *µ*L of RLT buffer supplied by RNeasy Mini Kit (Qiagen) for 5 min at room temperature. The mixture was transferred to an RNA Clean & Concentrator-5 spin column (Zymo Research) and further purified according to the manufacturer’s instructions.

### Gene expression analysis by reverse transcription quantitative PCR

RNA was isolated and converted to complementary DNA using SuperScript III First-Strand Synthesis SuperMix kit (Invitrogen) according to the manufacturer’s protocol. Reverse transcription quantitative PCR (RT-qPCR) was performed and quantified on CFX384 or CFX96 Real-Time PCR Detection System (Bio-Rad). Briefly, the five times diluted cDNA was used to perform RT-qPCR using SsoAdvanced Universal SYBR Green Supermix (Bio-Rad). All reactions were amplified at 50 °C for 2 min, 95 °C for 10 min, and 40 cycles of 95 °C for 10 s and 60 °C for 40 s, followed by a dissociation step to validate the PCR products. The data was analyzed using Bio-Rad CFX manager software. All reactions were run in technical triplicate. Target *C*t values were normalized to the internal housekeeping gene Ubiquitin. For MeRIP RT-qPCR, *C*t value was normalized to geometric mean of internal control (Input), including an internal housekeeping gene. Resulting normalized values were compared with target *C*t values using the 2^−ΔΔ*C*t^ method. The list of primers used in the study are listed in Supplementary Table S1.

### Phytohormone measurement

The extraction of phytohormones was performed as already described (Trapp et al. 2014). The compounds were quantified by HPLC-ESI-SRM, in a Thermo Fisher TQS-Altis Triple Quadrupole Mass Spectrometer coupled to a Thermo Scientific Vanquish MD HPLC system. The chromatographic separation was carried out in a UPLC column (Agilent Eclipse Plus C18, RRHD, 1.8 *μ*m, 2.1 × 50 mm), and the compounds were eluted using water (A) and acetonitrile (B) as mobile phase at 0.6 mL min^−1^ and in a gradient elution mode as following: 10% B for 0.5 min, 10% to 55% of B at 4.5 min, 55% to 100% of B at 4.7 min, 100% until 6.0 min, 100% to 10% of B at 6.1% and 10% until 8 min. The column was kept at 55 °C.

### Western blot

Two-week-old *pMTA:MTA-YFP* plants were harvested after 0, 3, and 6 DAD and flash frozen in liquid nitrogen. Five times SDS Loading dye was directly added to the ground powder and boiled at 85 °C for 10 min and later loaded on 10% (w/v) SDS-PAGE gel. Proteins were transferred to PVDF membrane and blocked with TBST-5% (w/v) milk for 2 h. Later blot was incubated with TBST-5% (w/v) milk containing anti-GFP (1:5,000; abcam) antibody overnight as primary antibody. After five washes, membrane was incubated with antirabbit secondary Ab (1:10,000, Promega) for 1 h. After five washes, membrane was developed using ECL Clarity Max solution (BioRad).

### Transcript stability time course

To measure mRNA stability, 2-wk-old seedlings were carefully transferred into ½ MS liquid media for 3 d in complete dark. Later, 30 *μ*g mL^−1^ of Actinomycin D (Sigma) and 10 *μ*g mL^−1^ cordycepin (Sigma) were added to the seedlings. After 1 h, the seedlings were transferred to perpetual light conditions for 3 and 6 h. Plants were harvested at 0, 3, and 6 h and flash frozen in liquid N_2_. Total RNA was extracted (described above) and RT-qPCR was performed as described above. The transcript amount at time 0 was treated as 100% and relative amounts of RNA were calculated at 24 h of inhibitor cocktail treatment.

### PSI data analysis

The plant phenotyping to measure various photosynthetic parameters was performed using PSI growth room (Photon Systems Instruments, Czech Republic).

The seeds of Arabidopsis Col-0 WT and *mta* seeds stratified at 4 °C in dark were then plated on ½ MS plates and grown in Percival at 16:8 light:dark for 7 d. The seedlings of similar root length were then transferred to PSI standard pots filled with same amount of SunGro soil mix, placed in PSI trays and then registered in PlantScreen system. The plants were grown at 22 °C with RH of 60% and 400 ppm CO_2_ at 16:8 light:dark for 14 d, after which the trays were removed and placed in complete dark for 3 d to induce DILS. After 6 d the trays were re-inserted into the PSI system and plants were imaged using florescence and RGB camera. Plants response to dark treatment was analyzed by image-based morphometric analysis and in-depth analysis of chlorophyll fluorescence kinetics after dark adaptation. Following equations were used to calculate variables in photochemistry: maximum quantum yield of PSII photochemistry: QYmax = *F*_v_/*F*_m_; the steady state nonphotochemical quenching: NPQ = (*F*_m_ − *Fʹ*_m_)/*Fʹ*_m_; relative fluorescence decline ratio: RFD_L_ss_ = (*F*_p_ − *F*t_L_ss_)/*F*t_L_ss_.

### Chloroplast ultrastructure by electron microscopy

The plant samples were fixed in first fixation buffer (2.5% (w/v) glutaraldehyde and 0.1 M phosphate buffer) at room temperature and then in second fixation buffer (2% (w/v) osmium tetraoxide and 0.1 M phosphate) at 4 °C until further processed. After staining with uranyl acetate, the samples were dehydrated through a gradient ethanol series. Later samples were embedded in Spurr’s resin and ultrathin sections were made using ultramicrotome. The sections, mounted on grids, were stained with uranyl acetate and lead citrate and photographed using TEM.

### Statistical analyses

Statistical significance was determined as mentioned in individual sections using GraphPad Prism 8.0 software. Data for quantitative analyses are presented as mean ± SD or mean ± SEM.

### Accession numbers

SAG12 (AT5G45890), SAG13 (AT2G29350), SAG113 (AT5G59220), SEN4 (AT4G30270), SARD1 (AT1G73805), WRKY6 (AT1G62300), WRKY53 (AT4G23810), OXI1 (AT3G25250), Trxh5(AT1G45145), CAB1 (AT1G29930), RBCS (AT1G67090), NYE1 (AT4G22920), GLK1 (AT2G20570), CP33B (AT2G35410), PIF4 (AT2G43010), ORE1/NAC2/NAC092 (AT5G39610), NAP(AT1G69490), TUB (AT5G62690), MTA (AT4G10760), MTB (AT4G09980), FIP37 (AT3G54170), VIR (AT3G05680), HAKAI (AT5G01160), ALKBH10B (AT4G02940), ECT1 (AT3G03950), ECT2 (AT3G13460), ECT3 (AT5G61020), ECT4 (AT1G55500), ECT6 (AT3G17330), ECT8 (AT1G79270), and CPSF30 (AT1G30460).

## Supplementary Material

kiad660_Supplementary_Data

## Data Availability

The RNA-seq data is submitted to NCBI with project no. PRJNA1025919.

## Supplementary data

The following materials are available in the online version of this article.

**Supplementary Figure S1**. Accelerated senescence in m^6^A mta and vir-1 mutants.

**Supplementary Figure S2**. Changes in chloroplast and photosynthesis in *mta* during DILS.

**Supplementary Figure S3**. Elevated DILS related hormone levels in *mta* mutant plants.

**Supplementary Figure S4**. Dynamic changes in transcripts of the m^6^A machinery upon DILS.

**Supplementary Table S1**. List of primers used for RT-qPCR analysis in this study.

## Dive Curated Terms

The following phenotypic, genotypic, and functional terms are of significance to the work described in this paper:

ABI3 Gramene: AT3G24650
ABI3 Araport: AT3G24650

WRKY22 Gramene: AT4G01250
WRKY22 Araport: AT4G01250

WRKY54 Gramene: AT2G40750
WRKY54 Araport: AT2G40750

WRKY70 Gramene: AT3G56400
WRKY70 Araport: AT3G56400

WRKY75 Gramene: AT5G13080
WRKY75 Araport: AT5G13080

MTA Gramene: AT4G10760
MTA Araport: AT4G10760

SAG21 Gramene: AT4G02380
SAG21 Araport: AT4G02380

NAP Gramene: AT1G69490
NAP Araport: AT1G69490

ETR2 Gramene: AT3G23150
ETR2 Araport: AT3G23150

JAZ7 Gramene: AT2G34600
JAZ7 Araport: AT2G34600

JAZ10 Gramene: AT5G13220
JAZ10 Araport: AT5G13220

RBCS1A Gramene: AT1G67090
RBCS1A Araport: AT1G67090
