# Supplementary material for: m6A RNA methylation counteracts dark-induced leaf senescence in Arabidopsis
Source: Plant Physiol. 2023 Dec 12;194(4):2663–78. doi: 10.1093/plphys/kiad660 (PMC10980409; doi:10.1093/plphys/kiad660)
Supplement: kiad660_Supplementary_Data [file kiad660_supplementary_data.pdf]

Supplemental Data

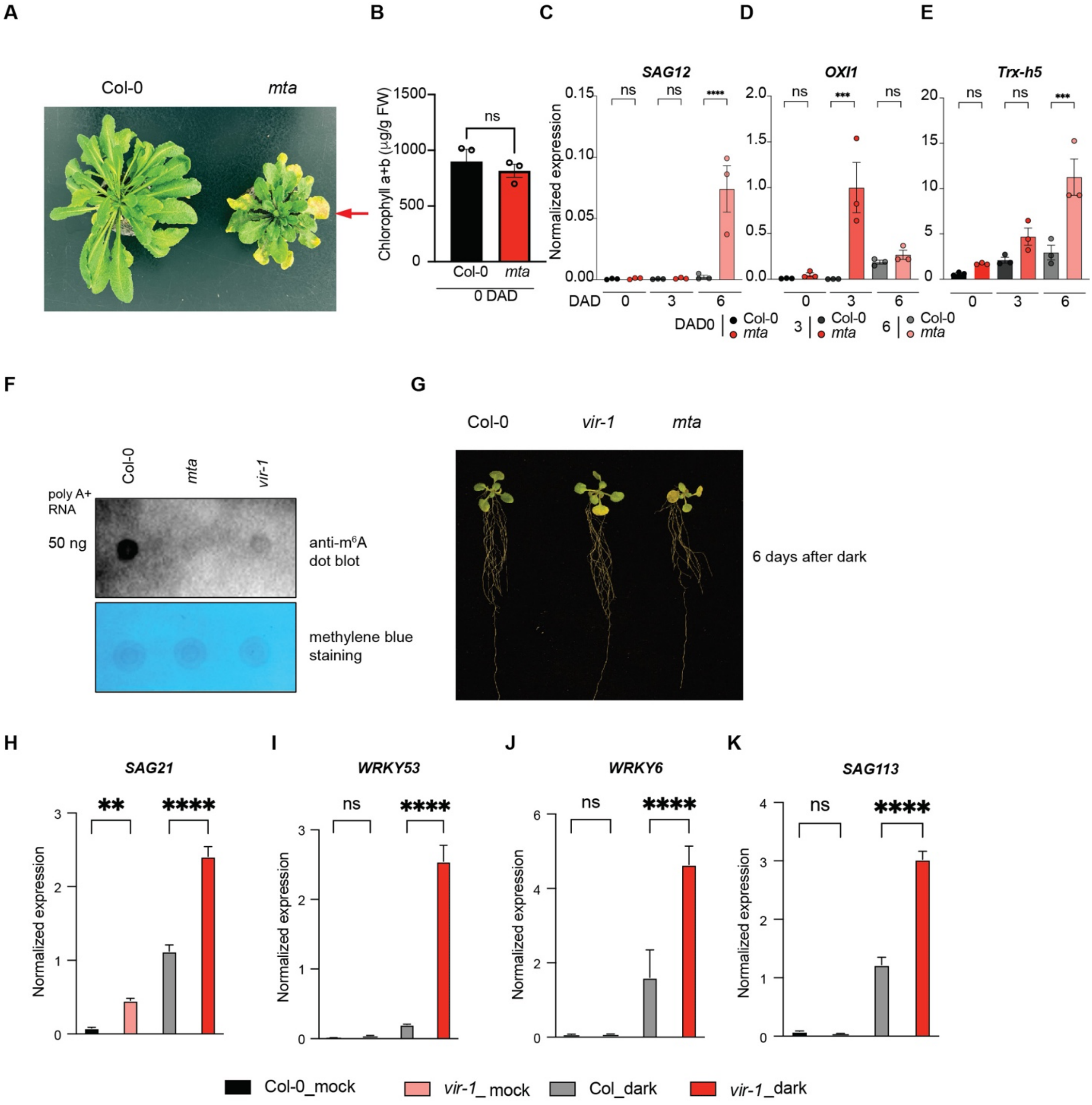

Supplemental Figure S1. Accelerated senescence in m^{6}A *mta* and *vir-1* mutants.
A, Phenotype of 6 week old Col-0 and mta plants grown in jiffy pots under short day conditions. Red arrow shows the leaf yellowing phenotype of *mta* mutant.
B, Comparison of the total chlorophyll content (chl a+b) of 2-week-old leaves between Col-0 and *mta* plants at 0 DAD. Values are presented as the mean ± SEM (n = 3) (two-tailed paired Student′s t-test, ns= non significant).
C, Transcript level of *SAG12*, a senescence marker gene in Arabidopsis
D-E, Transcript levels of ROS producing genes regulating senescence *OXI1* (D), *Trx-h5* (E) in Col-0 and *mta* seedlings upon dark treatment for 3 and 6 days.

F, Dot blots showing the global levels of m^6A in Col-0, *mta* and *vir-1* mutants**.** Methylene blue staining shows the loading control.

G, DILS phenotype in 2 week old plants grown on 1/2MS plates and kept in dark for 6 days

H-K, Expression levels of Arabidopsis senescence related marker genes *SAG21* (H), *WRKY53* (I), *WRKY6* (J) and *SAG113* (K) in Col-0 and *vir-1* seedlings dark treated for 6 days. The results shown are normalized to either *UBQ* or *Actin* expression as an internal control. $^{**}p < 0.01$, $^{***}p < 0.001$, $^{****}p < 0.0001$, multiple one-way ANOVA with Sidak's test. ns=not significant, DAD=days after dark.

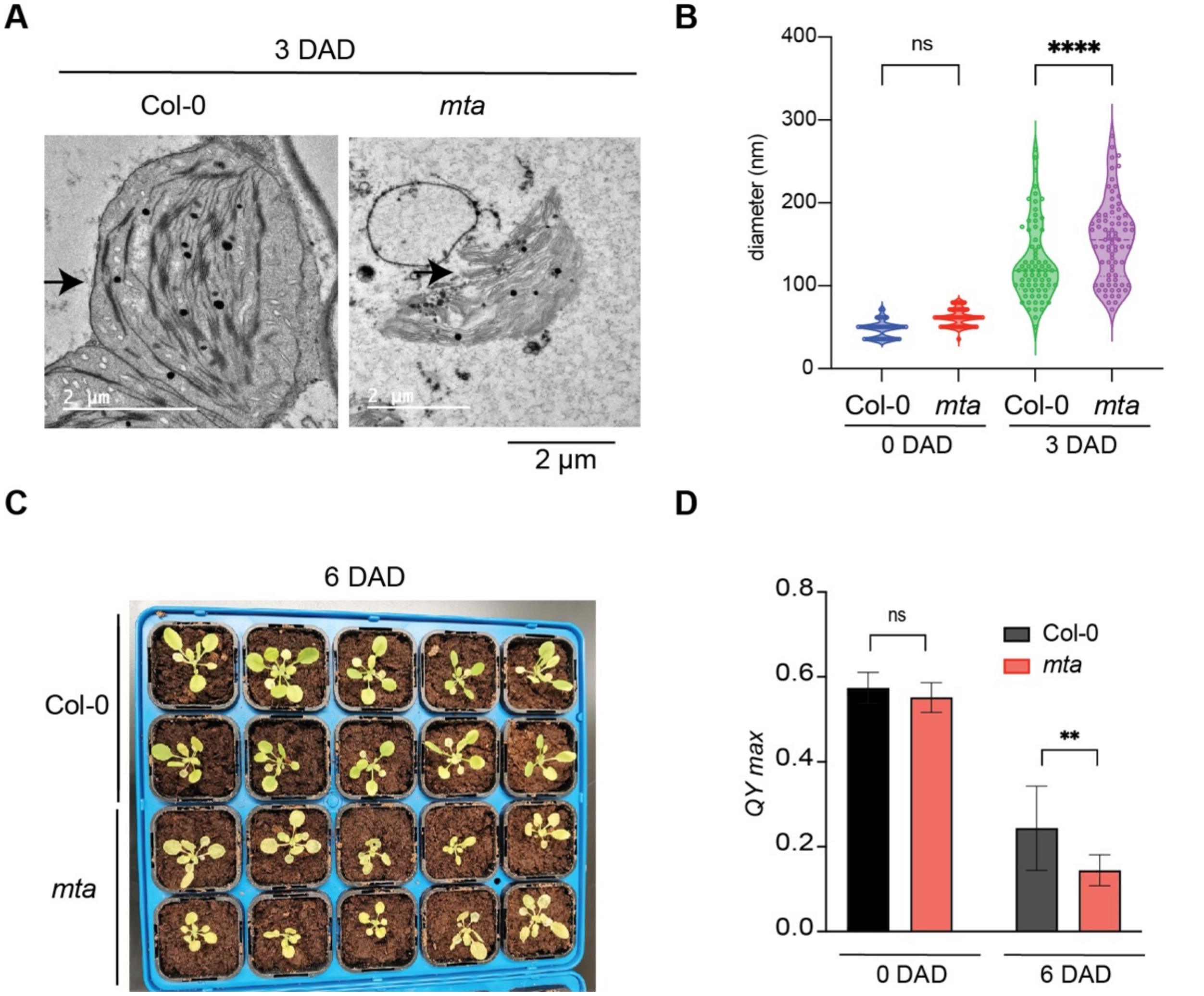

Supplemental Figure S2. Changes in chloroplast and photosynthesis in *mta* during DILS

A, TEM images showing chloroplast ultrastructure in Col-0 and *mta* at 3 DAD. Black arrows show the disintegration of chloroplast in *mta* while Col-0 has intact membrane (Scale bar=2 μm)

B, Plastoglobule quantification in control and dark treated chloroplasts. The data represents the diameter (in nm) of the plastoglobules calculated from TEM images. ****$p < 0.0001$, multiple one-way ANOVA with Sidak's test.

C, DILS phenotype in 2 week old plants grown on PSI compatible trays kept in dark for 6 days

D, QYmax showing the maximum quantum yield in Col-0 and *mta* plants before (0 DAD) and after (6 DAD) treatment. Values were counted by using Photon System Instruments, PSI system. Data presented here as mean ± SEM, **$p < 0.01$, multiple one-way ANOVA with Sidak's test. ns=not significant, DAD=days after dark.

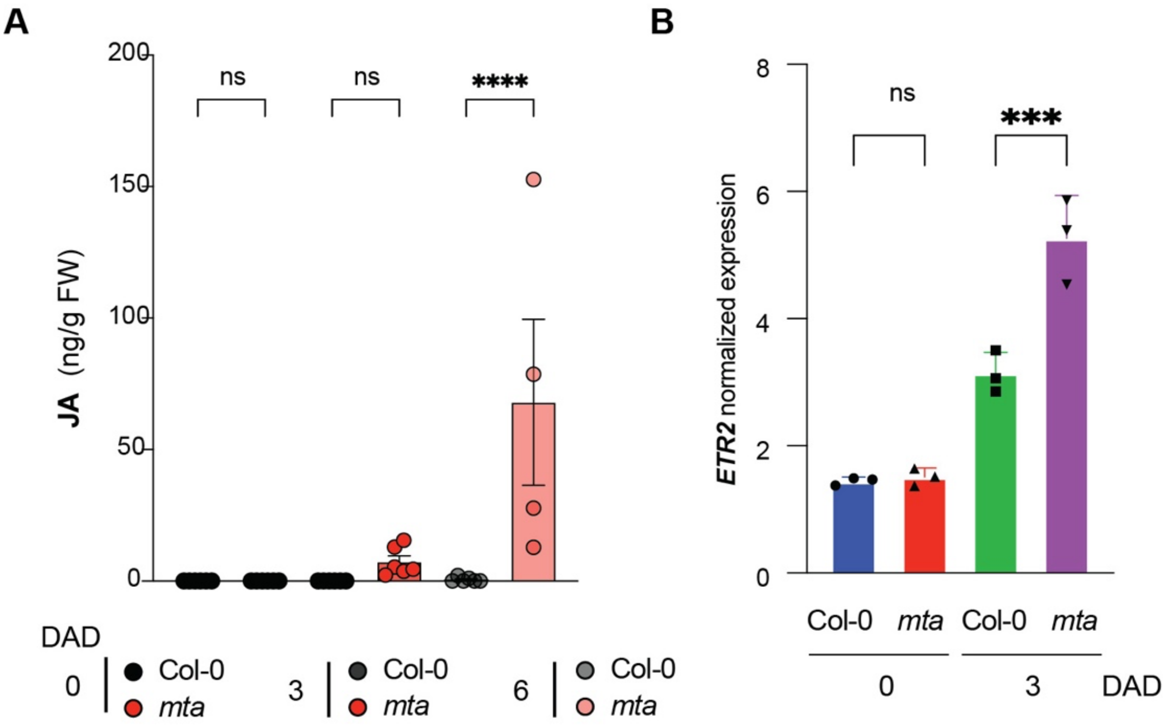

Supplemental Figure S3. Elevated DILS related hormone levels in *mta* mutant plants.

A, Quantification of Jasmonic acid (JA) levels (ng/g fresh weight) in Col-0 and *mta* seedlings dark treated for 3 and 6 days. Data is presented as mean ± SEM. ****$p < 0.0001$, multiple one-way ANOVA with Sidak's test. ns=not significant. JA= jasmonic acid

B, Transcript level of *ETR2*, a marker gene of ethylene signaling in Col-0 and *mta*.
Data is presented as mean ± SEM. ***$p < 0.001$, multiple one-way ANOVA with Sidak's test. DAD= Days after dark

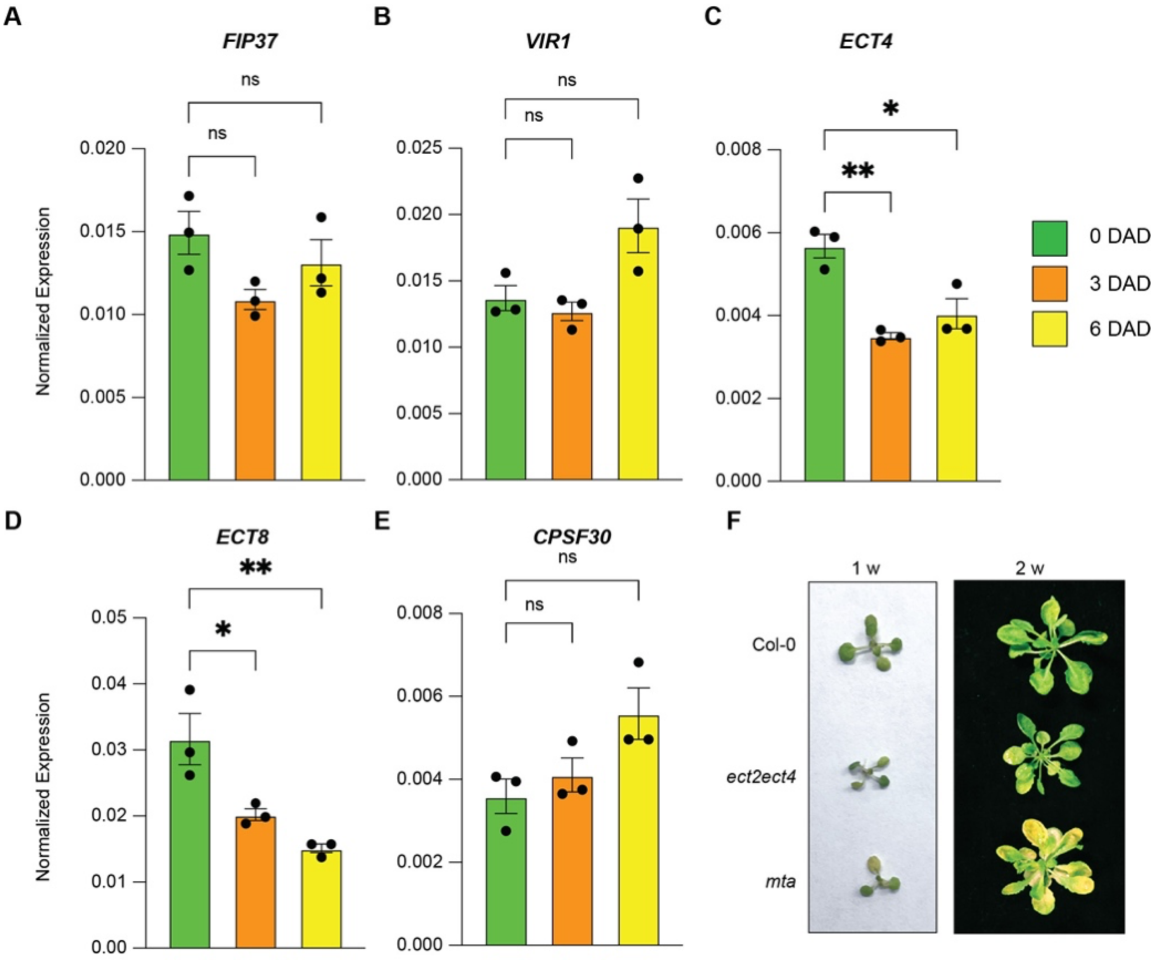

Supplemental Figure S4. Dynamic changes in transcripts of the m6A machinery upon DILS.

A-B, Expression levels of core Arabidopsis m6A writer genes *FIP37* (A) and *VIR1* (B) in Col-0 seedlings at 0, 3 and 6 DAD.

C-E, Expression levels of Arabidopsis m6A reader genes *ECT4* (C), *ECT8* (D) and *CPSF30* (E) in Col-0 seedlings at 0, 3 and 6 DAD. Data is presented as mean ± SEM. *$p < 0.05$, **$p < 0.01$, multiple one-way ANOVA with Sidak's test. ns=not significant, DAD=days after dark.

F, Comparison of leaf senescence between 1 and 2.5 week old seedlings of Col-0, *ect2ect4* and *mta* plants at 6 days after dark (DAD).

Supplemental Table S1. List of primers used for RT-qPCR analysis in this study.

Gene	Forward Primer	Reverse Primer
PIF4	CGACTCAGCCGATGGAGATGTT	GTTGTTGACTTTGCTGTCCCGC
SAG12	GCTTTCATGGCAAGACCACATAG	TGGATACGGCGAATCTACTAACG
CAB	CCAGAGGCATTCGCTGAGTTG	CCTTACCAGTGACGATGGCTTG
NYE1	GCAAGGATGGGCAAATAGG	CACCGCTTATGTGACAATGAAC
GLK1	GACACGCAAAAGGCATATCTAT	CTAAAATGATGGTGGTGGACAG
WRKY53	ATTCAAAGAAAAGAAAGATGTTACCAA	TCTCCAGCTAAAGACATCATCTTG
ORE1	GGGAAAATCACTTGTGGGTATG	GTTCTTAGCTGTTTGGGGAAGA
SAG21	ATCTTCCGACGTGGTTATGC	ATAACCGGTTTTGGGATCTG
JAZ10	CGCTCCTAAGCCTAAGTTCCA	TCGAAATCGCACCTTGAATA
WRKY6	GCAACAGCAACAACAGAACAA	TGCCTTGGTACTATCGTCTCC
SAG29	GCCACCAGGGAGAAAAGG	CCACGAAATGTGTTACCATTAGAA
SAG113	CCATGGCTGTTCCCATGTA	AAGCTACGCGCCATTGAC
RBCS	CGCTCCTTTCAACGGACTTA	AGTAATGTCGTTGTTAGCCTTGC
CPSF30	ACAACTCGCTTCTTTGCTTTATC	GGATTCACACCCTTTGCTTTC
ECT1	TTCCCTGTGAAGTGGCATATC	GTCCCATGCTCCAGATTCAC
ECT2	TCGAGGAATCAAAACTACCGC	TCTCCCTGTGCTACCATACTG
ECT4	GTACCTGCGACTAGGAATCAAA	AGTAACCCTGAGCTGCATAAC
ECT8	AGATCGTCCTAAAACACCGC	TCGCCTTCTCTGTTTCATCC
MTB	GTACTGTGTTTCAGCGTTCC	TTCTGAGTCGAACCATAAGGAG
VIR	ACGCAAGTCCAGCCTTACTATCAC	CGGTCACTTAATAGAGCCTGAATGG
ALKBH10B	CAGCTTGGTGTCCCCATCTT	GGCTGTGAGTATTCACCCTCT
FIP37	CTCAACTCAAGCCAGCGTCA	CTTGGTGCCCAATCTCCTCA
HAKAI	CAGCAGGTTAACCGGGGTAG	TTTTTGGCACGGTTTTGGCT
MTA	TGCCGTCTTCAGTCTGGTTC	ATAGCGTCAGCCATGTCGAG
NAP	ACG TGT TCG CTG GCT CAT TT	CCG AAC CAA CTA GAC TCC GAA TCA
CP33B	CCTGGAGACACTCGTCACAA	AAACAACCCGAGCTGAGACC
OXI1	TCTCTTCCGCTTCACCAGTT	CCTAACGACCACCAATCGAC
Trx-h5	TGAATTGCAAGCTGTTGCTC	GCAGAAGCTACAAGACCACC
UBQ	TGGACGCTTCAGTCTGTGTG	TTCTTAGGCATAGCGGCGAG
